# Proteomic Analysis Reveals Enzymes for β-D-Glucan Formation and Degradation in *Levilactobacillus brevis* TMW 1.2112

**DOI:** 10.3390/ijms23063393

**Published:** 2022-03-21

**Authors:** Julia A. Bockwoldt, Chen Meng, Christina Ludwig, Michael Kupetz, Matthias A. Ehrmann

**Affiliations:** 1 Lehrstuhl für Mikrobiologie, Technische Universität München, 85354 Freising, Germany; julia.bockwoldt@tum.de; 2 Bayerisches Zentrum für Biomolekulare Massenspektrometrie (BayBioMS), Technische Universität München, 85354 Freising, Germany; chen.meng@tum.de (C.M.); tina.ludwig@tum.de (C.L.); 3 Lehrstuhl für Brau- und Getränketechnologie, Technische Universität München, 85354 Freising, Germany; michael.kupetz@tum.de

**Keywords:** *Levilactobacillus brevis* TMW 1.2112, β-glucan, exopolysaccharide, glycosyltransferase, glycosyl hydrolase, moonlighting proteins, secretome, proteome

## Abstract

Bacterial exopolysaccharide (EPS) formation is crucial for biofilm formation, for protection against environmental factors, or as storage compounds. EPSs produced by lactic acid bacteria (LAB) are appropriate for applications in food fermentation or the pharmaceutical industry, yet the dynamics of formation and degradation thereof are poorly described. This study focuses on carbohydrate active enzymes, including glycosyl transferases (GT) and glycoside hydrolases (GH), and their roles in the formation and potential degradation of *O2*-substituted (1,3)-β-D-glucan of *Levilactobacillus* (*L*.) *brevis* TMW 1.2112. The fermentation broth of *L*. *brevis* TMW 1.2112 was analyzed for changes in viscosity, β-glucan, and D-glucose concentrations during the exponential, stationary, and early death phases. While the viscosity reached its maximum during the stationary phase and subsequently decreased, the β-glucan concentration only increased to a plateau. Results were correlated with secretome and proteome data to identify involved enzymes and pathways. The suggested pathway for β-glucan biosynthesis involved a β-1,3 glucan synthase (GT2) and enzymes from maltose phosphorylase (MP) operons. The decreased viscosity appeared to be associated with cell lysis as the β-glucan concentration did not decrease, most likely due to missing extracellular carbohydrate active enzymes. In addition, an operon was discovered containing known moonlighting genes, all of which were detected in both proteome and secretome samples.

## 1. Introduction

Exopolysaccharide (EPS) formation of lactic acid bacteria (LAB) has been massively studied for structural and sensory effects in the food industry and as drug delivery agents, bio-absorbents, and probiotics in the pharma industry [1,2,3,4,5,6,7,8,9,10,11,12]. The advantage of EPSs from LABs is that they are generally recognized as safe (GRAS) and could be used under in vitro or in vivo conditions [4]. EPSs are high-molecular-weight polymers, either secreted into the surrounding environment or acting as capsular polysaccharides attached to the cell surfaces (CPS) [13]. The EPSs produced by LAB are classified into homopolysaccharides (HoPSs) such as glucans and fructans, formed by repeating units of the same monosaccharide or heteropolysaccharides (HePS), which are mainly composed of D-glucose, D-galactose, and L-rhamnose. Most HoPSs (e.g., dextran, mutan, inulin, or levan) are polymerized extracellularly by glucansucrases or fructansucrases, whereas HePSs and β-glucans (consisting exclusively of D-glucose monomers) are formed intracellularly by transmembrane glycosyltransferases from nucleotide-activated sugars and released to the extracellular environment during polymerization [2,14,15,16,17]. The purposes of EPS and CPS formation are often described as protectors against biotic and abiotic stress (e.g., temperature, pH, osmotic stress, or antimicrobial compounds) and as important agents in biofilm formation and cell–cell interaction [18,19,20,21,22].

On the contrary, although a decrease in EPS was observed in many studies by physical processes of the culture or enzymatic activity, the functions of EPS degradation by LAB remain unclear [23,24,25,26,27,28,29]. In general, the enzymatic hydrolysis of polysaccharides is performed by enzymes of the glycoside hydrolases (GH) families. GHs belong to the numerical classification EC 3.2.1.- and possess hydrolytic activity to glycosidic bonds of carbohydrates and non-carbohydrate fractions [30,31]. However, some EPS-producing LABs do not even possess hydrolytic enzymes enabling EPS degradation [4,32].

*Levilactobacillus* (*L*.) *brevis* TMW 1.2112 is a heterofermentative LAB isolated from spoiled beer due to capsular *O2*-substituted (1,3)-β-D-glucan formation [33,34]. *L*. *brevis* TMW 1.2112 was applied to in-situ-enriched EPS sourdough, to investigate the structural and sensory characteristics of the dough and the subsequent baked goods [35,36]. Furthermore, the physiological effects of isolated β-glucan from *L. brevis* TMW 1.2112 as well as plant-based and yeast β-glucans were analyzed in a comparative study [37]. Previous studies have described the polymerization of β-glucan by transmembrane glycosyltransferase-2 (Gtf-2) family members called β-1,3 glucan synthases (GT2), e.g., in *Pediococcus* (*Pe.*) *parvulus*, *Pediococcus* (*Pe.*) *claussenii*, *Paucilactobacillus* (*Pa.*) *suebicus*, and *L*. *brevis* sp. [35,38,39,40,41,42,43]. The Gtf-2 enzymes belong to the numerical classification EC 2.4.-, which comprises several glycosyl transferases (GTs). Enzymes of this class catalyze the transfer of sugar moieties from activated donor molecules to acceptor molecules, resulting in the formation of glycosidic bonds [44,45]. Moreover, Fraunhofer et al. (2018) postulated a putative pathway for β-glucan biosynthesis by *L*. *brevis* TMW 1.2112 through the genome sequence [46].

Homologies to the β-glucan and Gtf-2 of LAB were observed for the β-1,3 β-1,2 glucan capsule and glycosyltransferase Tts branched from *Streptococcus* (*S*.) *pneumoniae* 37 [47]. An immunoagglutination test using *S. pneumoniae* serotype 37 antibodies enables the identification of β-glucan capsules of LAB and was positive for *L. brevis* TMW 1.2112 [34], *Pe. claussenii* TMW 2.340 [35], *Oenococcus oeni* [42], and *Pediococcus damnosus* [48]. However, proteins involved in the attachment of the β-1,3-glucan capsule to the cell surface of LAB are so far undescribed. It has been discussed that the LytR-Cps2A-Psr (LCP) protein family and the Wzy pathways could be involved in polysaccharide attachment to the peptidoglycan of Gram-positive bacteria [49,50,51,52,53]. Still, the knowledge about LCPs in Lactobacilli is limited. Furthermore, moonlighting proteins are known to overtake multiple functions based on their cellular position, e.g., moonlighting proteins from commensal lactobacilli were described as acting in adhesion processes [54,55,56,57]. LCP and moonlighting proteins might interact with the CPS of *L*. *brevis* TMW 1.2112 regarding attachment and adhesion processes, but such phenomena have not yet been described.

Although a decrease in the β-glucan amount and viscosity effects in sourdough and culture broth fermented by *L*. *brevis* TMW 1.2112 was observed, the responsible factors are unknown [34,36]. To avoid weak solubility and possible structural changes due to the multistage isolation processes of isolated β-glucan, the in vivo expression of GTs and GHs during fermentation of *L*. *brevis* TMW 1.2112 can be studied by proteomic analysis [58,59]. Consequently, we aimed to identify the enzymes involved in β-glucan formation and the subsequent presumed degradation by the regulation of differentially expressed proteins during the exponential, stationary, and early death phases. We hypothesize that the observed changes in viscosity and β-glucan concentration are reflected in the proteome and correlate with the presence of relevant enzymes (GTs, GHs, and β-glucan biosynthesis). This study revealed a pathway for the biosynthesis of (1,3)-β-D-glucan of *L. brevis* TMW 1.2112 and demonstrated a lack of enzymatic activity for the polymer utilization as energy sources.

## 2. Results

### 2.1. Growth Characteristics of L. brevis TMW 1.2112 and β-Glucan Content in Culture Broth

**Figure 1:**
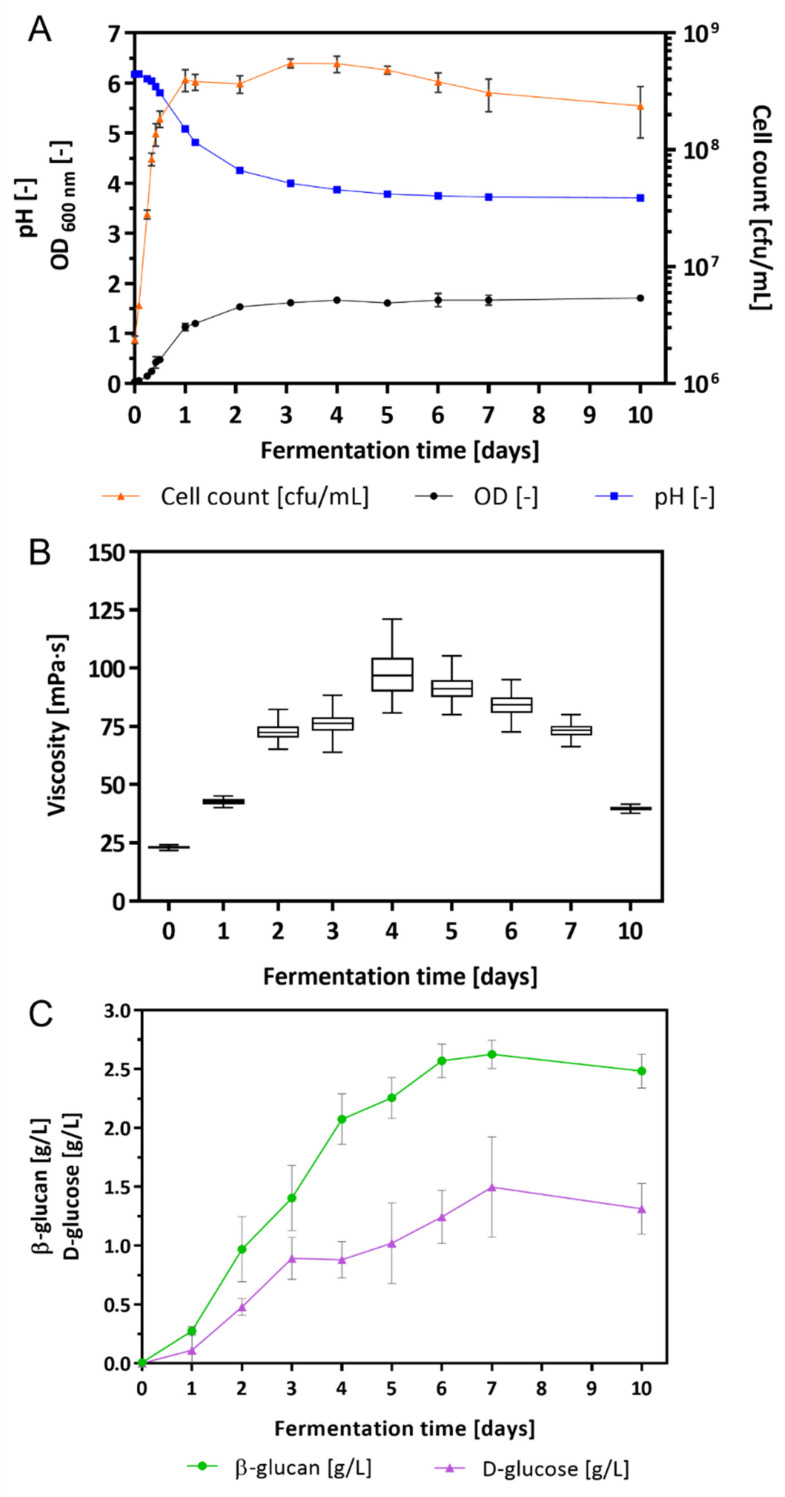
Growth characteristics of the fermentation broth: (**A**) cell count in cfu/mL, OD, and pH, (**B**) changes in the viscosity, and (**C**) β-glucan and D-glucose concentration in culture supernatants. Values are mean values of four-fold biological replicates including standard deviations.

*L. brevis* TMW 1.2112 was cultivated in a chemically defined medium (CDM) for 10 days. During the fermentation process, growth parameters, e.g., cell count, pH value, viscosity, and the amount of β-glucan and D-glucose, were analyzed. After inoculation, the cell growth entered the exponential phase immediately and reached the stationary phase within 24 h (Figure 1A). During fermentation, the cell count increased with its maximum after 3 days with 5.5 × 10^8^ cfu (colony forming units)/mL. After 5 days, the cell count decreased to 4.8 × 10^8^ cfu/mL. The pH value decreased from the initial pH 6.2 to 5.1 after 24 h and to 3.7 after 7 days. Values of the optical density (OD) increased within 2 days in fermentation to 1.5 and was 1.7 after 10 days.

The changes in viscosity were determined by a rotational viscometer once per day (Figure 1B). After inoculation of the CDM with *L. brevis* TMW 1.2112, the viscosity of the culture broth was 22.8 ± 0.9 mPa⸱s increasing continuously to 97.2 ± 26.4 mPa⸱s within 4 days. An increase in viscosity resulted in an increase in variance because of the heterogeneous viscoelastic characteristic of the culture broth. With progress in fermentation the viscosity values and the corresponding variances decreased again to finally 39.5 ± 2.5 mPa⸱s. Within 10 days the viscosity of the culture broth increased significantly (until 4 days) and subsequently approached towards the initial value.

In addition, the amount of β-glucan and D-glucose (monomeric units of β-glucan) was measured by an immunological and an enzymatical method (Figure 1C). The initial concentrations of both compounds were 0.00 g/L in the supernatant. Within 4 days, the β-glucan concentration was 2.08 ± 0.19 g/L and increased further to 2.63 ± 0.10 g/L within 7 days. A similar trend was observed for the D-glucose with an increase in the concentration after 4 and 7 days, resulting in 0.88 ± 0.13 g/L and 1.50 ± 0.37 g/L, respectively. After 4 days, the increase in the β-glucan became slower, and the accumulation of D-glucose slowed down after 3 days. At the end of fermentation (10 days), the concentrations of both compounds declined marginally to 2.48 ± 0.13 g/L (β-glucan) and 1.31 ± 0.19 g/L (D-glucose). The chemically defined medium with only maltose as a carbon source was used as a blank for both assays.

### 2.2. Glycosyl Transferases (GT) and Glycoside Hydrolases (GH) in L. brevis TMW 1.2112

The genome of *L. brevis* TMW 1.2112 c (GenBank accession No.: CP016797) [34,60] was additionally annotated by RAST and eggNOG-Mapper for clusters of orthologous groups (COG) and functionality, resulting in 2184 annotated proteins [61,62,63]. The genome sequence comprised 49 glycosyltransferases (GT; EC 2.4.-) and glycoside hydrolases (GH; EC 3.2.1.-), which were identified and characterized by several databases (NCBI BLASTx, UniProt, CAZy (Carbohydrate-Active enZYmes) and eggNOG-Mapper) for their protein classification and molecular functions (Table 1) [30,61,62,63,64,65]. SignalP-5.0 predicted six of the 49 annotated GHs and GTs as secreted enzymes and mostly were associated with cell wall biosynthesis [66]. Of note, the endo-β-1,3-glucanase (AZI09_02135), β-1,3-glucosidase BglB (AZI09_02170), and Gtf-2 proteins (AZI09_03685, AZI09_12985, AZI09_07565, AZI09_06585, AZI09_04045, AZI09_12875, and AZI09_10605) are enzymes potentially involved in the formation and degradation of β-glucan [15,41]. The transmembrane Gtf-2 (AZI09_12770), which is encoded on the plasmid pl12112-4 (GenBank accession No.: CP016801) of *L. brevis* TMW 1.2112, was considered particularly relevant [38,40].

### 2.3. Proteomic Analysis

#### 2.3.1. Secretome: Protein Secretion from Exponential to Early Death Phases

**Figure 2:**
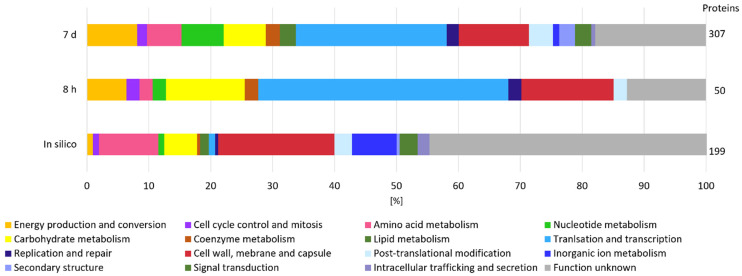
Secretome analysis by COG classification. The in silico analyzed secretome was compared with samples from the beginning (8 h) and end (7 days) of fermentation of at least three out of four replicates. On the right, the total numbers of identified gene locus IDs are stated.

Considering that β-glucan is an extracellular EPS and that its degradation is most likely initiated in the extracellular environment by GHs, we measured the secretome of *L. brevis* TMW 1.2112 in the exponential phase (8 h) and at the end of fermentation (7 days) to study the functions and roles of proteins associated with EPS degradation. We identified more proteins by their gene locus IDs at the end of fermentation (307 detected proteins) compared to the exponential phase (50 proteins detected). The proteins reproducibly detected in at least three out of four replicates were retained in the downstream analysis. The in-silico analysis using SignalP-5.0 predicted 199 secreted proteins, including endo-β-1,3-glucanase (AZI09_02135). Figure 2 shows the cluster of orthologous groups (COG) and functional characterization of the in-silico secretome and both time points (8 h and 7 days). Energy production and conversion, carbohydrate metabolism, and coenzyme metabolism generated larger shares within the analyzed samples compared to the in silico secretome, and the share of proteins with unknown function was reduced at the same time. Proteins of the carbohydrate metabolism group made up 13% during the exponential phase and only 7% during the death phase. Approximately 25% of the detected proteins in both samples were previously assigned as hypothetically secreted proteins.

**Figure 3:**
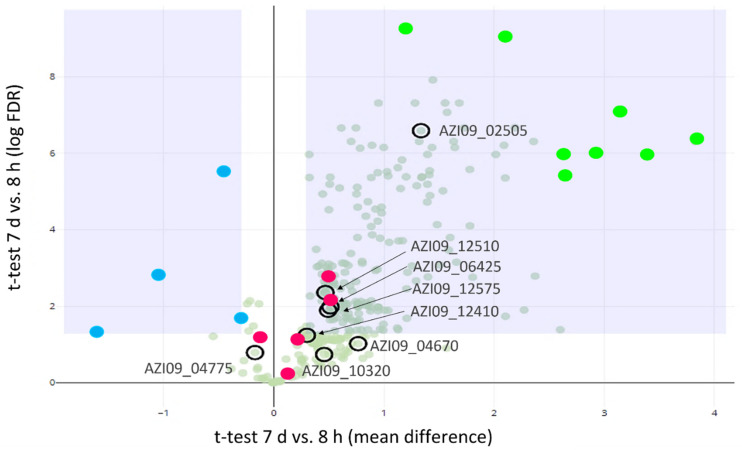
Proteomics analysis of secreted proteins. Volcano plot for the differential abundance analysis of 8 h vs. 7 d secretomes: GHs and GTs (**encircled**), proteins with higher abundance at 8 h (**blue dots**), proteins with higher abundance at 7 days (**light green dots**), and potential moonlighting proteins (**red dots**).

A Student *t*-test was used to identify proteins that were differentially secreted between 8 h and 7 days (Figure 3). In total, 8 GHs and GTs were detected in our experiment, including two GH65 family proteins (AZI09_10320 and AZI09_04670) maltose phosphorylases (hereinafter referred as MP), two GH73 family proteins N-acetylmuramoyl-L-alanine amidase (AZI09_02505 and AZI09_04775), the GT51 family protein penicillin-binding protein (AZI09_06425), two GT8 family proteins (AZI09_12410 and AZI09_12575), and GH31 family protein α-glucosidase (AZI09_12510). Enzymes of the GT8 family have been described as acting as nucleotide-diphospho-sugar glycosyltransferases [67]. The endo-β-1,3-glucanase (AZI09_02135), which was assumed to be involved in β-glucan degradation, was not detected. All GHs and GTs were detected after 7 days, except GH73 N-acetylmuramoyl-L-alanine amidase (AZI09_04775), which was also present after 8 h.

The secretion of four proteins were higher after 8 h compared to 7 days. Those four proteins were annotated as phosphoketolase (AZI09_09710), NlpC P60 family (AZI09_02850 and AZI09_01220), and LSU ribosomal protein L3p (L3e) (AZI09_03445) (Figure 3, blue dots). After 7 days, the number of secreted proteins increased significantly, including the ABC transporters (AZI09_01955 and AZI09_00135), ribosomal proteins (AZI09_03485 and AZI09_05315), and uncharacterized proteins (AZI09_10715 and AZI09_11665) (Figure 3, green dots).

Moreover, several proteins, which were previously described as moonlighting proteins in bacteria were detected, such as enolase (AZI09_08765), triose-phosphat isomerase (TPI) (AZI09_08770), phosphoglycerate kinase (PGK) (AZI09_08775), glyceraldehyde-3-phosphate dehydrogenase (GADPH) (AZI09_08780), and elongation factor (EF) Tu (AZI09_05335). Those proteins were detected within both samples, except TPI, which was only detected after 7 days (Figure 3, red dots) [68].

Additionally, the secretome was analyzed for proteins involved in β-glucan biosynthesis and proteins associated with polysaccharide capsules and stress response. This resulted in the detection of β-phosphoglucomutase (β-PGM) (AZI09_04665), MFS maltose transporter, MalT (AZI09_10325), UTP--glucose-1-phosphate uridylyltransferase (UGP) (AZI09_08865), and glucokinase (AZI09_07205) after 7 days. The previous named enzymes are members of the postulated pathway for β-glucan formation by *L. brevis* TMW 1.2112 [46]. According to capsular polysaccharide attachment, two proteins of LytR-CpsA-Psr (AZI09_03640 and AZI09_03715) were detected after 7 days [49,50,51,52]. An arginine deiminase (ADI) (AZI09_01860) was present after 8 h and 7 days in the secretome. Several proteins identified by the secretome analysis are common intracellular proteins. After 7 days, the number increased, which could be due to cell lysis according to the growth phase.

The number of uncharacterized proteins accounted for 47% of the identified secretome, by only two proteins of the secretome after 8 h (Figure S1). The top ten proteins of the 8 h secretome-comprised cell wall and the cell-surface-related proteins were expressed. Further, only AZI09_01220, which codes for N1pC/P60 proteins, a family of cell-wall peptidases, was present within the 8 h sample, but not after 7 days. The highest expression (iBAQ intensity [69]) within both secretome samples was an uncharacterized protein (AZI09_12405) with a molecular weight (MW) of 49.9 kDa. Bioinformatic characterization (SWISSMODEL and PredictProtein) showed that this protein might be a cell wall or a surface layer protein [70,71]. Moreover, after 7 days in fermentation, the protein with the second highest expression was an ABC transporter binding protein (AZI09_01995) with an MW of 25.1 kDa and a putative signal peptide.

#### 2.3.2. Cell Lysate: Changes in Protein Expression over Fermentation Time

**Figure 4:**
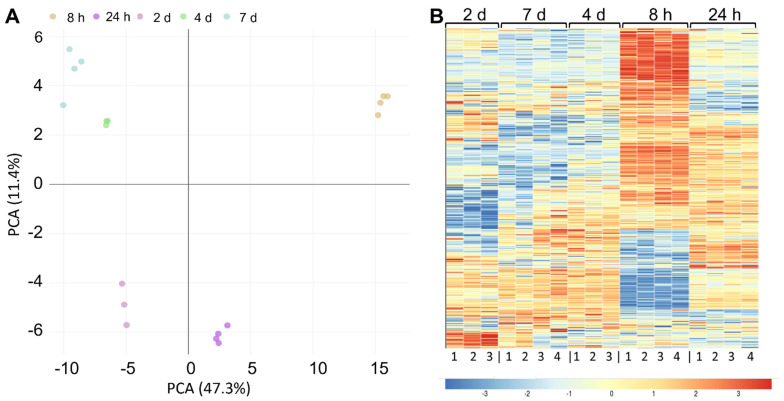
Proteomics analysis of cellular proteins over fermentation. (**A**) Principal component analysis (PCA) of the five fermentation time points. (**B**) Heat map using Pearson correlation displaying the protein abundance at five different time points (8 h, 24 h, 2 days, 3 days, and 7 days) after the hierarchical clustering of 1641 proteins. Three- to four-fold biological replicates were used for the analysis. The changes in enzyme expression are depicted by color intensity, as indicated below the figure.

To examine how protein expression changes during fermentation, we measured the proteome of *L. brevis* TMW 1.2112 at five time points during fermentation, in the exponential (8 h), stationary (24 h to 4 d), and early death phases (7 d). *L. brevis* TMW 1.2112 genome contains 2537 predicted protein coding genes, and 1641 proteins were identified by proteomic analysis. This corresponds to a proteome coverage of approximately 65%, which is in the range of other label-free quantitative proteomic analyses of LABs [72,73]. Principal component analysis (PCA) revealed a high similarity between biological replicates (Figure 4A). The samples of 4 days and 7 days in fermentation clustered together, while the sample of 8 h was the most distinguishable from other time points. To continue the global comparison of the five time points, a heat map was generated, and the samples were hierarchically clustered using Pearson correlation distance (Figure 4B). Most differences were obtained for the 8 h time point. The clusters of 7 days and 4 days were highly similar.

**Figure 5:**
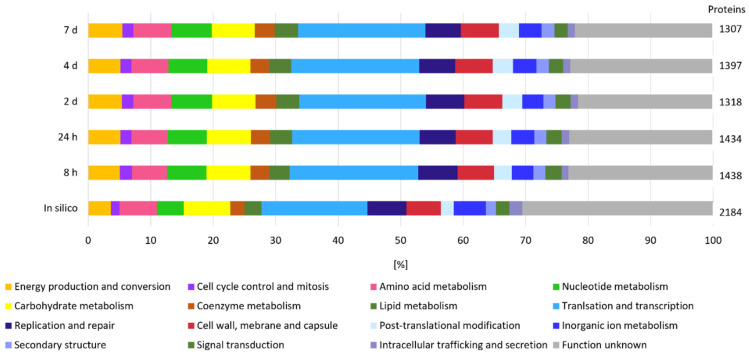
Cellular proteome analysis by COG classification. The in silico proteome was compared at five time points (8 h, 24 h, 2 days, 3 days, and 7 days) in at least three out of four or two out of three replicates.

The proteome analyzed in silico counted 2184 annotated proteins, which were used for COG and functional characterization (Figure 5). COG prediction of the in silico proteome showed a pattern similar to that of the expressed proteins at the five time points. A total of 30% of the expressed proteins could not be categorized (unknown function). Between the five time points (8 h, 24 h, 2 days, 3 days, and 7 days), no significant difference was observed according to the distribution of the groups. The COG analyses of the expressed proteins accounted for translation and transcription ~20% each: ~7% were related to carbohydrate metabolism and approximately 5% were clustered for energy production and conversion. Proteins of the last two groups are considered to be related to polysaccharide formation and degradation, among others.

Fraunhofer, Jakob, and Vogel (2018) postulated a putative pathway for the β-glucan biosynthesis based on analyses of the *L*. *brevis* TMW 1.2112 genome sequence. The β-glucan biosynthesis from maltose as a substrate included a transporter for maltose, MP, glucokinase, phosphoglucomutase, UGP, UDP kinase or nucleoside-diphosphate kinase (NDPK), and Gtf-2 [46]. Several pathway members have been measured in our proteomics data, e.g., MalT (transporter) (AZI09_10325), MPs (AZI09_04670 and AZI09_10320), glucokinase (AZI09_07205), β-PGM (AZI09_04665 and AZI09_02330), UGP (AZI09_08865), NDPK (AZI09_05450), and Gtf-2 family proteins (AZI09_04045, AZI09_12875, and AZI09_12770). According to β-glucan degradation, β-1,3-glucosidase BglB (AZI09_02170) was detected during the whole fermentation. The endo-β-1,3-glucanase (AZI09_02135) was only expressed at 4 days (late stationary phase). Genes associated with polysaccharide encapsulation, stress response, and moonlighting proteins as previously described with respect to the secretome were also present within the cell lysate samples.

#### 2.3.3. Cell Lysate: Expression of GHs and GTs and Their Correlation with Viscosity, β-Glucan, and D-Glucose Concentrations

**Figure 6:**
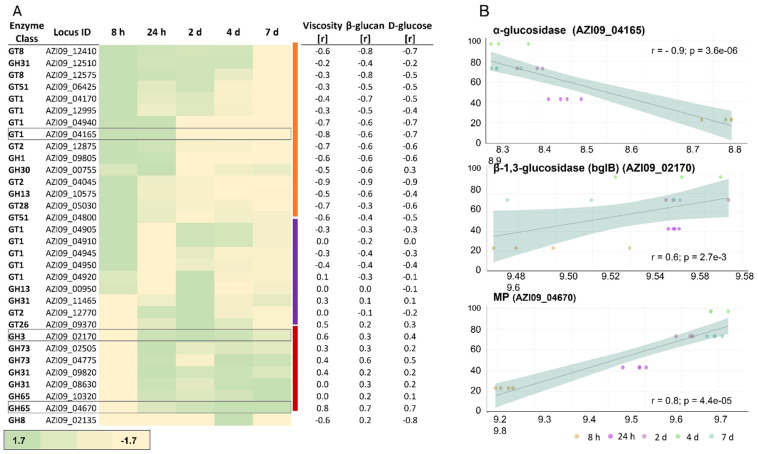
Expression of GHs and GTs in the cell lysate. (**A**) Heat map of the expressed enzymes over time. The three clusters of proteins are indicated by the color bar on the right (orange, purple, and red). Relative fold change of protein expression compared to the mean depicted by color intensity, as stated below the figure (a range between −1.7-fold and 1.7-fold). Correlation coefficient values with viscosity, β-glucan, and D-glucose are listed next to the specific GHs and GTs. (**B**) The correlation of the expression of α-glucosidase AZI09_04165, β-1,3-glucosidase (bglB) AZI09_02170, and the MP AZI09_04670 with viscosity, including correlation coefficient values (r) and *p*-values (*p*).

In total, 32 of the previously described 49 GHs and GTs were detected in our proteomic data. We manually assigned these proteins into three clusters according to their expression over time (Figure 6A).

The first cluster (orange cluster, Figure 6A) included enzymes that were increased during the early growth phase and decreased in the further course of fermentation. The second cluster (purple cluster, Figure 6A) contained enzymes with higher expressions during the early growth phase and/or the beginning of the stationary phase. The third cluster (red cluster, Figure 6A) represented enzymes that increased towards the end of fermentation. Additionally, the correlation between protein expression and viscosity, β-glucan, and D-glucose concentration were examined (Figure 6B). During exponential and the early stationary phase, the GT1 family protein 1,2-diacylglycerol 3-glycosyltransferase, *pimA* (AZI09_04165) was more abundant compared to the late stationary and death phases, which also resulted in an inverse correlation with changes in viscosity (Figure 6B), β-glucan, and D-glucose. Members of this enzyme family catalyze the transfer of sugar moieties from nucleotide-sugar donors to membrane-associated acceptor substrates and are involved in plasma membrane synthesis [74]. Gtf-2 family proteins AZI09_12875 and AZI09_04045 were assigned to the second cluster, and Gtf-2 AZI09_12770 was grouped into the third cluster. The expression of the two MPs (GH65) AZI09_04670 and AZI09_10320 increased with progress in fermentation (8 h to 7 days). However, only the MP AZI09_04670 correlated strongly (r ≥ 0.8) with changes in viscosity and D-glucose concentration, while MP AZI09_10320 showed no correlations [75]. Furthermore, the β-1,3-glucosidase (AZI09_02170) as a member of the GH3 family was expressed on a constant level after 24 h, with medium to low correlations with the growth characteristics. The GH8 family protein endo-β-1,3-glucanase (AZI09_02135) was only expressed after 4 days when the viscosity of the fermentation broth decreased continuously (Figure 1).

#### 2.3.4. Correlation between Protein Expression and Growth Characteristics

In addition to the correlations of GHs and GTs described in the previous section, the expression patterns of all other proteins were correlated with viscosity, β-glucan, and D-glucose to reveal potential novel proteins with a function related to β-glucan formation or degradation. Proteins of the pathway for β-glucan biosynthesis resulted in moderate to strong correlations (0.6 ≤ r ≤ 0.8), e.g., β-PGM (AZI09_04665) and NDPK (AZI09_05450), with all growth characteristics. The sequence of MP (AZI09_04670) is encoded downstream to β-PGM and showed similar correlation coefficients. Moonlighting-associated proteins, e.g., enolase (AZI09_08765), TPI (AZI09_08770), PGK (AZI09_08775), and GADPH (AZI09_08780), showed low to strong correlations (0.4 ≤ r ≤ 0.8) with all three characteristics. Strong inverse correlations (r ≤ −0.7) were observed for LytR-CpsA-Psr (AZI09_03640) and ADI (AZI09_01860) with D-glucose, and only moderate inverse correlations with β-glucan were observed. The results of the correlation are listed in a Supplementary Excel file (Table S1).

**Figure 7:**
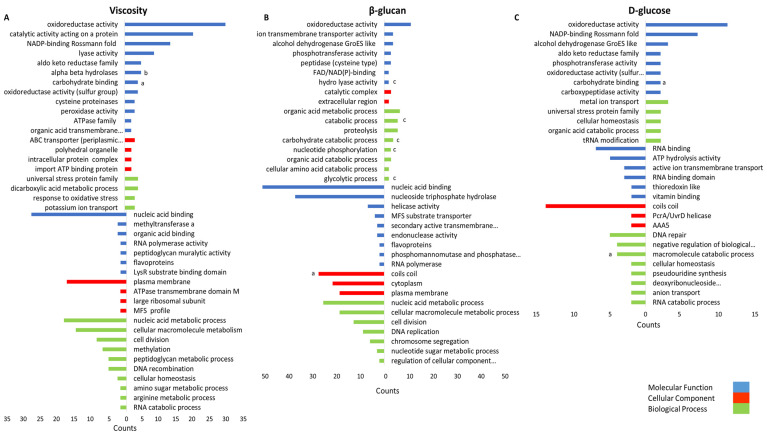
Gene set overrepresentation analysis of proteins correlating with (**A**) viscosity, (**B**) β-glucan content, and (**C**) D-glucose content. Top bar charts indicated the positively and bottom bar chart the negatively correlating proteins with corresponding GO terms (*y*-axis). The *x*-axis represents the gene counts. GO terms comprising proteins of interest are indicated by (a) GHs and GTs; (b) β-glucan-biosynthesis-associated proteins (other than GHs and GTs), and (c) moonlighting-associated protein enolase (AZI09_08765).

To obtain a comprehensive understanding of the gene expression during β-glucan biosynthesis or degradation, we performed an overrepresentation analysis using proteins significantly correlated with these parameters (r ≥ 0.7 and r ≤ −0.7). In total 118, 433, and 404 proteins correlated with D-glucose, β-glucan, and viscosity, respectively. The resulting gene ontology (GO) terms were categorized in three groups: molecular function (MF), cellular component (CC), and biological process (BP) (Figure 7). The GO term oxidoreductase activity (MF) correlated with all three growth characteristics. Among others, nucleic acid binding (BP), cell division (BP), and other terms associated with cell growth correlated inversely with viscosity and β-glucan. Carbohydrate-metabolism-associated terms, e.g., glycolytic process (BP), carbohydrate process (BP), and carbohydrate binding (MF), correlated positively with the analyzed characteristics, while the macromolecular catabolic process (BP) correlated inversely with D-glucose concentration. However, only three GHs and GTs were identified within the terms: the MP (GH65) AZI09_04670, part of the gene set of the term carbohydrate binding, the GH8 family protein endo-β-1,3-glucanase (AZI09_02135) of the macromolecular catabolic process, and the GT8 family protein (AZI09_12410) of coils coils. Moreover, enolase (AZI09_08765), a putative moonlighting protein and an enzyme of glycolysis, was identified in BP terms correlating with the β-glucan concentration, e.g., the organic acid metabolic process, the cellular amino acid catabolic process, and the glycolytic process. The results of the overrepresentation analysis are listed in a Supplementary Excel file (Table S2).

## 3. Discussion

In this study, proteomics based on state-of-the-art LC-MS/MS was used to analyze the biosynthesis and potential degradation of *O2*-substituted (1,3)-β-D-glucan by *L. brevis* TMW 1.2112 on a molecular level. The focus of the study was to identify correlations between the expression of carbohydrate-active enzymes and changes in the EPS state (viscosity, β-glucan, and D-glucose concentration) by biosynthesis and degradation. Since β-glucan forms a polysaccharide capsule (CPS) around the cells, proteomic data of both the cell lysate and secretome were analyzed to actively identify the involved GHs and GTs [35,38,76].

**Figure 8:**
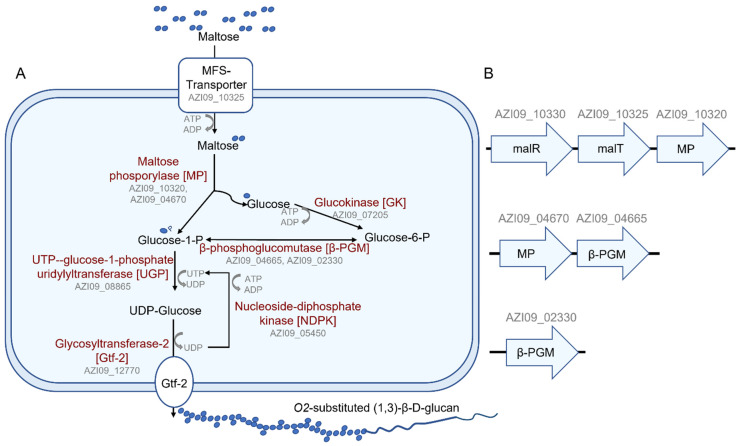
β-glucan biosynthesis of *L. brevis* TMW 1.2112. (**A**) Metabolic pathway of the β-glucan biosynthesis, as suggested from genomic data and proteomic analysis. (**B**) Suggested maltose operons: malR = transcriptional regulator, MP = maltose phosphorylase, malT = MFS maltose transporter and β-PGM = β-phosphoglucomutase.

Pathways for the biosynthesis of β-glucan by *L. brevis* TMW 1.2112 from several mono- and disaccharides have been postulated [46]. With maltose as a sole energy source, the proteomic data were analyzed for enzymes related to this putative pathway. In summary, all proteins described in the putative pathway were identified within the cell lysate, and some were identified within the secretome (Figure 8A). Transporters for maltose uptake in LAB, e.g., the maltose ABC transporter or the MFS maltose transporter, have been described in *Lactiplantibacillus* (*La*.) *plantarum* or *Fructilactobacillus* (*F.*) *sanfranciscensis*, respectively [77,78]. Our analyses resulted in the MFS maltose transporter, MalT (AZI09_10325), expressed within the cell lysate and secretome (7 days). The ORF of MalT was next to the ORF of the MP (AZI09_10320) and the ORF of the transcriptional regulator MalR (AZI09_10330), resulting in one operon. These proteins were detected at all time points of the cell lysate and after 7 days within the secretome. In addition, the ORFs of the MP AZI09_04670 and β-PGM (AZI09_04665) could be associated with a second operon, and a second β-PGM (AZI09_02330) was also identified (Figure 8B). Two maltose operons were described for *F. sanfranciscensis*: one contains an MP (*mapA*) and β-PGM and is induced by the presence of maltose acting as a major catabolic enzyme, and the second includes, in addition to an MP (*mapB*) and β-PGM, a permease and epimerase. The mapB system is under the control of a constitutive promotor and a microorganism, and only the mapA system usually has a prolonged lag phase [79]. The cell growth of *L. brevis* TMW 1.2112 entered the exponential phase immediately without a lag phase, which could also be due to the faster utilization of the free amino acids of the CDM.

The β-glucan biosynthesis began simultaneously with the cell growth. While the cell count slowed down after one day, the β-glucan concentration continued to increase significantly, leading to the assumption that β-glucan formation was independent from the cell count and accumulated over time. This behavior has been observed in other β-glucan-forming LABs [43,80,81]. Proteome analysis revealed three expressed GT2 family proteins: two chromosomal encoded GT2 proteins (AZI09_04045 and AZI09_12875), both significantly more abundant during the exponential phase, inversely correlated with viscosity and D-glucose concentration, and a plasmid-encoded GT2 protein (AZI09_12770), which was most likely constantly expressed. The transmembrane β-1,3 glucan synthase (GT2 family) is described as the key enzyme in the bacterial β-glucan biosynthesis [15,41,46,48]. The GT2 family comprises thousands of sequences with at least 12 distinct GT functions, making a functional prediction by homology difficult [82]. However, Llamas-Arriba et al. (2018) compared the nucleotide sequences of 13 β-1,3 glucan synthases of *Pediococcus* spp., *Oenococcus oeni*, and *Lactobacillus* spp., including *L. brevis* TMW 1.2112 (AZI09_12770). The study revealed that β-1,3 glucan synthase sequences are highly conserved and mostly plasmid-encoded [41,83]. Sequence alignments of the two chromosomally encoded GT2s) from *L. brevis* TMW 1.2112 resulted in low identities (data not shown). Consequently, only AZI09_12770 is a β-1,3 glucan synthase and responsible for β-glucan biosynthesis.

The start of β-glucan production during the exponential phase has been observed in *Pe. parvulus* spp. and *Pe. damnosus* IOEB8801 [81,84]. In addition, *Pa. suebicus* CUPV221 started β-glucan production during the stationary phase [43]. Nevertheless, the strains have one thing in common with *L. brevis* TMW 1.2112: the continuous increase in β-glucan concentration even up to 14 days and the lack of its decrease [43,81,84]. What differed, however, was the viscosity of *L. brevis* TMW 1.2112 cultures, as it decreased significantly after 4 days, unlike the viscosities of *Pediococcus* strains and *Pa. suebicus*, which increased steadily until 7 days or more and remained high [43,85]. The decrease in the viscosity and heterogeneous viscoelastic characteristics might be associated with the enzymatical or physical effects degrading high-molecular EPS (≥9.6 × 10^6^ Da) into smaller fractions (at least 6.6 × 10^6^ Da), which could still be detected by ELISA [86]. A broad detection range of the immunological assay could also result in an overestimation of the polymer concentration. The endo-β-1,3-glucanase (AZI09_02135) was, despite the identification of a signal peptide, only detected within the cell lysate during the stationary phase (4 days). Sequence analysis revealed two stop-codon mutations within the last 30 amino acids of the 367 amino acid sequence of the endo-β-1,3-glucanase (AZI09_02135), which might affect enzyme activity or secretion. Furthermore, the overrepresentation analysis resulted in an inverse correlation for D-glucose and the term macromolecular catabolic process. This in turn means that changes in the D-glucose concentration were not a result of endo-β-1,3-glucanase activity. Most of the GHs were identified as intracellular enzymes, such as β-1,3-glucosidase (BglB) (AZI09_02170), which was also not present in the secretome, though it was expressed during the fermentation period. Moreover, the arginine deiminase (ADI), known for its role in the defense against acidic stress, was also expressed during fermentation [87,88]. This could lead to the assumption that a low pH value might have interfered with the viscosity, but physical effects due to self-produced organic acids appeared to be unlikely, as this was so far not observed for other β-glucan-producing LABs such as pediococci [43,84,85]. Further, capsular EPSs are described as protectors against environmental stress such as acidity [34,89,90]. Nevertheless, the fermentation broth underwent structural changes during fermentation, which could not be explained by the enzymes listed in Table 1. The secretome contained so far uncharacterized enzymes that may affected the viscosity, since many proteins could not be clearly categorized. Though changes in the heterogeneous character of the viscosity might be attributed to the formation of a β-glucan-cell-network, lysed cells reduced the network integrity during the late stationary and early death phases, and this in turn might have led to the reduced viscosity [34,35]. Autolysis, which is strain-dependent and preferentially triggered under stress conditions or in late growth phases, is a well-known phenomenon in lactic acid bacteria [91,92]. Moreover, the increased number of proteins in the secretome after 7 days compared to 8 h could also be explained by the autolysis of *L. brevis* TMW 1.2112 cells.

Potential β-1,3-glucan degradation could be represented by an increase in the D-glucose concentration as the monomeric unit [15,38,76,80]. An increase in the D-glucose concentration was detected but with a similar curve progression as the β-glucan concentration, which means that the release of D-glucose started already during the exponential phase and simultaneously with β-glucan biosynthesis. This in turn means that it is rather a product of maltose utilization than of β-glucan degradation. Glucose secretion after the phosphorolytic cleavage of maltose is described in other lactobacilli [93]. The utilization of maltose strains and the subsequent glucose release by beer-spoiling *L. brevis* has been described [94]. In conclusion, β-glucan is not used as a storage compound by *L. brevis* TMW 1.2112. The CPS therefore might have mostly a protective function according to the strain’s origin from beer with present ethanol, low pH, or antimicrobial hop compounds [34,95].

The proteins responsible for the attachment of the β-1,3-glucan capsule to the cell surface of LAB have not been described. Members of the LytR-Cps2A-Psr (LCP) protein family are discussed for their role in the attachment of polysaccharides to the peptidoglycan of Gram-positive bacteria [49,50,51,52]. According to sequence analyses, *L. brevis* TMW 1.2112 possess three LCP family proteins, two of which were constantly expressed according to the cell lysate data and after 7 days within the secretome. Both proteins (AZI09_03640 and AZI09_03715) were identified as BrpA (Biofilm regulatory protein A) proteins, with AZI09_03715 containing a putative signal peptide. The knowledge about *Lactobacilli* LCPs is currently limited. Nevertheless, it was assumed that the Wzy pathways could be involved in the coupling of CPS to the cell surface peptidoglycan [52,53,96]. Genes of the Wzy pathway were not detected within the proteomic data or the genome sequence, but CDSs for a chain-length determining protein (AZI09_03645) and an EPS biosynthesis protein (AZI09_03650) are encoded next to BrpA (AZI09_03640). However, neither protein was detected by MS. Further experiments are necessary to identify specific enzymes involved in the attachment of the capsules to cell surfaces.

**Figure 9:**
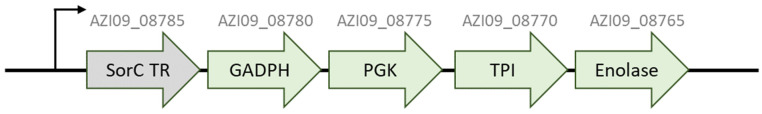
Operon of putative moonlighting proteins. GADPH (glyceraldehyde-3-phosphate dehydrogenase), PGK (phophoglycerate kinase), TPI (triose-phosphat isomerase), and enolase.

Several proteins that have been described as acting as moonlighting proteins in other bacteria emerge regularly within the proteomic data of the cell lysate and secretome, including an enolase (AZI09_08765), triose-phosphate isomerase (TPI) (AZI09_08770), phosphoglycerate kinase (PGK) (AZI09_08775), glyceraldehyde-3-phosphate dehydrogenase (GADPH) (AZI09_08780), and elongation factor Tu (EF-Tu) (AZI09_05335). Most moonlighting proteins are primarily enzymes of the glycolytic and metabolic pathways or molecular chaperones [54,68] and overtake multiple functions based on their cellular position, for example, when released into the extracellular milieu [54,68]. For example, the enolase is grouped in terms associated with metabolic processes, which is not surprising, as enolases are involved in the glycolysis. Interestingly, all proteins except TPI (AZI09_08770) were detected after 8 h within the secretome, even though these proteins are usually intracellular proteins. The moonlighting proteins GADPHs, EF-Tu, and the enolases of commensal lactobacilli have been described as acting in adhesion processes and might contribute to probiotic traits [54,55,56,57]. Further, four of these so-called moonlighting genes were located within an operon including a transcriptional regulator (AZI09_08785), which was also detected within the cell lysate samples (Figure 9). An operon of moonlighting proteins was previously found in *Staphyloccocus aureus* [97], and while moonlighting proteins of pathogens and probiotics are generally described to be highly conserved [54], it could be assumed that these genes, which are also grouped with an operon, might have moonlighting functions in *L. brevis* TMW 1.2112. However, the effect of these genes, e.g., on the β-glucan-cell-network, on the adhesion to surfaces, or even on probiotic actions, requires further studies to identify their specific roles in the extracellular milieu.

In the present study, we have shown that, over a period of fermentation lasting 10 days, the glucose concentration and the amount of β-glucan in the supernatant increased, but the viscosity decreased. The biosynthesis of β-glucan is closely linked to the maltose metabolism via an MP. Genes encoding enzymes involved in maltose utilization are organized in three operons. The continuous increase in glucose is probably due to the phosporolytic cleavage of maltose rather than the degradation of the β-glucan to the monomer. Thus, we assume that β-glucan is not used as a storage substance or degraded by the cells for further utilization. In addition, a limitation of ELISA differentiating between high- and low-molecular-weight β-glucan might cause misinterpretations of the concentrations and cause its actual decrease to evade detection. There are probably several reasons for the decrease in viscosity. The only carbohydrate active enzyme that strongly correlated with viscosity, β-glucan, and D-glucose concentrations was an MP, and none of the enzymes with a β-glycosidic bond preference as the endo-β-1,3-glucanase or β-1,3-glucosidase (BglB). Aside from an enzymatic cleavage by GHs secreted or released by partial autolysis, destruction of the β-glucan cell network due to a detachment of capsular β-glucan bound to cell surfaces also seems plausible. Moreover, two new study approaches focusing the extracellular polysaccharide encapsulation and cell–cell adhesion were identified with respect to the LCP protein family and moonlighting proteins, respectively. Furthermore, the secretome contained proteins with potential degradable functions on β-glucan with respect to the viscosity that have hitherto been uncharacterized. This study provides important insight into growth characteristics associated with the β-glucan formation of *L. brevis* TMW 1.2112 and creates a basis for future investigations of EPS-forming LAB in which the role of growth characteristics and EPS biosynthesis is studied.

## 4. Materials and Methods

### 4.1. Strain, Medium, and Growth Conditions

*L. brevis* TMW 1.2112 is a LAB isolated from wheat beer. The strain was cultivated in a modified chemically defined medium (CDM). The CDM (pH 6.2) was mixed from a 970 mL base medium with a 10 mL vitamin solution, a 10 mL metal solution, and a 10 mL nucleic acid base solution, as previously described by Otto et al. (1983) and Sánchez et al. (2008) with further modifications. The base medium contained (quantities per liter of distilled water) 20 g of maltose, 2.5 g of K_2_HPO_4_ · 3 H_2_O (VWR International, Radnor, PA, USA), 3 g of KH_2_PO_4_ (VWR International, Radnor, PA, USA), 0.6 g of di-ammonium hydrogen citrate (Carl Roth GmbH & Co. KG, Karlsruhe, Germany), 1 g of sodium acetate (Carl Roth GmbH & Co. KG, Karlsruhe, Germany), 0.25 g of cysteine-HCl (Carl Roth GmbH & Co. KG, Karlsruhe, Germany), 5 g of casamino acids (MP Biomedicals GmbH, Germany), and 1 g of Tween 80^®^ (Merck KGaA, Darmstadt, Germany). The vitamin solution (pH 7.0) contained (quantities per liter of distilled water) 100 mg of nicotinic acid, 100 mg of thiamine-HCl, 100 mg of riboflavin, 100 mg of pantothenic acid, 1 g of aminobenzoic acid, 1 g of D-biotin, 100 mg of folic acid, 100 mg of vitamin B_12_, 500 mg of orotic acid, 500 mg of thymidine, 500 mg of inosine, 250 mg of lipoic acid, 500 mg of pyridoxine (all chemicals were purchased from Sigma-Aldrich, St. Louis, MO, USA, except for folic acid (Carl Roth GmbH & Co. KG, Karlsruhe, Germany), and 100 mg of vitamin B_12_ (AppliChem GmbH, Darmstadt, Germany). The metal solution contained (quantities per liter of distilled water) 20 g of MgCl_2_ · 6 H_2_O (Sigma-Aldrich, St. Louis, MO, USA), 5 g of CaCl_2_ · 2 H_2_O (Carl Roth GmbH & Co. KG, Karlsruhe, Germany), 0.5 g of FeCI_2_ · 4 H_2_O (Fluka Chemie GmbH, Buchs, Switzerland), 0.5 g of ZnCl_2_ (Merck KGaA, Darmstadt, Germany), and 0.25 g of CoCl_2_ · 6 H_2_O (Sigma-Aldrich, St. Louis, MO, USA). The nucleic acid base solution contained (quantities per 10 mL 0.1 M NaOH) 10 mg of adenine sulfate (SERVA Electrophoresis GmbH, Germany), 10 mg of uracil (Sigma-Aldrich, St. Louis, MO, USA), 10 mg of xanthine (Sigma-Aldrich, St. Louis, MO, USA), and 10 mg of guanine (Fluka Chemie GmbH, Buchs, Switzerland) [98,99].

### 4.2. Fermentation and Monitoring of Cell Growth

To investigate the formation and degradation of β-glucan by *L. brevis* TMW 1.2112 intra- and extracellularly, two sets, each with four biological replicates, were inoculated with an initial OD_600 nm_ of 0.05, aliquoted (50 mL) and incubated at 30 °C as static cultures in 50 mL reaction tubes (Sarstedt AG & Co., Darmstadt, Germany). Aliquots of the first set were used for proteomic analyses, while the second set was used for viscosity analyses. Cell growth was monitored for 10 days based on OD measurements, cell count analysis, and changes in the pH values. The colony forming units (cfu) were analyzed with inoculated mMRS agar plates incubated at 30 °C for 48 h before counting as previously described [35]. All experiments were carried out using four biological replicates.

### 4.3. Proteomic Analysis

#### 4.3.1. Proteomic Sample Preparation

The *L. brevis* TMW 1.2112 fermentation broths (50 mL) were centrifuged at 17,000× *g* for 30 min at 4 °C. For cellular proteome measurements, the cell pellets were processed, while the supernatants were collected for secretome analyses.

*Cellular proteomes:* Bacterial cell pellets were washed once with a 50 mL saline solution (Ringer tablets, Merck KGaA, Darmstadt, Germany). After a second centrifugation step, the cell pellets were shock-frozen in liquid nitrogen and stored at −80 °C. For cell lysis, between 300 and 1600 μL of lysis buffer (8 M urea (Gerbu Biotechnik GmbH, Heidelberg, Germany), 100 mM NH_4_HCO_3_ (Sigma-Aldrich, St. Louis, MO, USA), 1 mM dithiothreitol (DTT, Gerbu Biotechnik GmbH, Heidelberg, Germany) in water, pH 8.0), and acid-washed glass beads (Ø 2.85–3.45 mm, Carl Roth GmbH & Co. KG, Karlsruhe, Germany) were added, depending on the previous OD of the culture broth. The cell pellet of the 50 mL culture broth with an OD_600_ of 1 was resuspended in 1 mL of lysis buffer. The cells were disrupted with a benchtop homogenizer (FastPrep^®^-24 MP, MP Biomedical Inc., Eschwege, Germany) in five cycles of 45 s each at 5 m⸱s^−1^. Between the cycles, the samples were stored on ice to cool (1 min). Cell lysates were collected after centrifugation (17,000× *g*, 10 min, 4 °C), and the total protein concentrations were determined using the Coomassie (Bradford) protein assay kit (ThermoFisher Scientific, Waltham, MA, USA) according to the manufacturer’s protocol. For each sample, 15 µg of protein extract was reduced with 10 mM DTT (Carl Roth GmbH & Co. KG, Karlsruhe, Germany) and carbamidomethylated with 55 mM chloroacetamide (CAA, Merck KGaA, Darmstadt, Germany). Subsequently, proteins were digested twice with 0.15 µg of trypsin (Roche Deutschland Holding GmbH, Penzberg, Germany), first for 2 h and then overnight at 37 °C. Digested peptide samples were desalted and resuspended in 2% acetonitrile (VWR International, Radnor, PA, USA), 98% H_2_O, and 0.1% formic acid (CARLO ERBA Reagents GmbH, Emmendingen, Germany) for a final concentration of 0.1 µg/µL.

*Secretomes:* For secretome analyses, 500 µL of the fermentation medium were mixed at 2:1 with NuPAGE™ LDS Sample Buffer (4×) (ThermoFisher Scientific, Waltham, MA, USA) and heated for 10 min at 70 °C. In-gel trypsin digestion was performed according to standard procedures [100]. Briefly, the samples were run on a NuPAGE™ 4–12% Bis-Tris Protein Gel (Thermofisher Scientific, Waltham, MA, USA) for 3 min. Subsequently, the still not size-separated single protein band per sample was cut out of the gel, reduced (50 mM DTT), alkylated (55 mm CAA), and digested overnight with trypsin (trypsin-gold, Promega, Madison, WI, USA). The sample was freshly re-suspended before MS measurement in 25 µL of 2% acetonitrile and 0.1 formic acid, and 3 µL were injected into the mass spectrometer per measurement.

#### 4.3.2. LC-MS/MS Measurements

LC-MS/MS measurements were performed on an Ultimate 3000 RSLCnano system coupled to a Q-Exactive HF-X mass spectrometer (Thermo Fisher Scientific, Waltham, MA, USA). For proteome analyses, ca. 0.2 µg of peptides were delivered to a trap column (ReproSil-pur C18-AQ, 5 μm, 20 mm × 75 μm, self-packed, Dr. Maisch GmbH, Ammerbuch-Entringen, Germany) at a flow rate of 5 μL/min in HPLC grade water with 0.1% formic acid. After 10 min of loading, peptides were transferred to an analytical column (ReproSil Gold C18-AQ, 3 μm, 450 mm × 75 μm, self-packed, Dr. Maisch GmbH, Ammerbuch-Entringen, Germany) and separated using a 50 min gradient from 4% to 32% of Solvent B (0.1% formic acid in acetonitrile and 5% (*v*/*v*) DMSO (Sigma-Aldrich, St. Louis, MO, USA)) at a 300 nL/min flow rate. Both nanoLC solvents (solvent A = 0.1% formic acid in HPLC grade water and 5% (*v*/*v*) DMSO) contained 5% DMSO to boost MS intensity. The Q-Exactive HF-X mass spectrometer was operated in data-dependent acquisition (DDA) and positive ionization mode. MS1 spectra (360–1300 m/z) were recorded at a resolution of 60,000 using an automatic gain control (AGC) target value of 3e6 and a maximum injection time (maxIT) of 45 ms. Up to 18 peptide precursors were selected for fragmentation for full proteome analyses. Only precursors with a charge state from 2 to 6 were selected, and a dynamic exclusion of 25 s was enabled. Peptide fragmentation was performed using higher energy collision induced dissociation (HCD) and a normalized collision energy (NCE) of 26%. The precursor isolation window width was set to 1.3 m/z. The MS2 resolution was 15,000 with an automatic gain control (AGC) target value of 1e5 and a maximum injection time (maxIT) of 25 ms.

#### 4.3.3. LC-MS/MS Data Analysis

Peptide identification and quantification were performed using the software MaxQuant (version 1.6.3.4) with its built-in search engine Andromeda [101,102]. MS2 spectra were searched against the proteome database of *L. brevis* TMW 1.2112 (GenBank accession No.: CP016797), including 2537 coding sequences supplemented with common contaminants (built-in option in MaxQuant). Trypsin/P was specified as a proteolytic enzyme. Precursor tolerance was set to 4.5 ppm, and the fragment ion tolerance was 20 ppm. Results were adjusted to a 1% false discovery rate (FDR) on a peptide spectrum match (PSM) level and a protein level employing a target-decoy approach using reversed protein sequences. The minimal peptide length was defined as 7 amino acids, and the “match-between-run” function was enabled (matching time window: 0.7 min; alignment window: 20 min). Carbamidomethylated cysteine was set as a fixed modification and oxidation of methionine and N-terminal protein acetylation as variable modifications. To compare relative protein abundances in the cell lysate time course experiment, label-free quantification (LFQ) was used. The LFQ assumes that the overall protein abundance across samples is comparable. This assumption is clearly violated in the secretome experiment; therefore, we used intensity-based absolute quantification (iBAQ) in this analysis [103]. Each sample type was analyzed in biological triplicates (at minimum).

#### 4.3.4. Statistical Analysis of Proteomics Data

In the downstream analysis, the proteins identified as “only identified by site”, “reversed”, and “potential contaminants” were removed first. The iBAQ intensites (for the secretome experiment) were centered across samples based on the proteins shared between 8 h and 7 days. The LFQ intensity was already well normalized and therefore not further changed. The intensities were logarithm-transformed on base 10. Proteins identified in less than 3 replicates out of 4 were excluded from the statistical analysis, and the remaining missing values were imputed using the lower detection limit method [104]. The missing values of a protein expression were replaced by the constant, which was half of lowest detected values. If the imputed value was higher than the 15% quantile of all the detected values, the missing value was replaced by the 15% quantile. The rationale for this is based on the fact that the missing values are more likely to result from low abundant proteins.

A Student *t*-test was used to identify proteins that were significantly differentially expressed between 8 h and 7 days in the secretome experiment. For the cell lysate time course experiment, Pearson correlation analysis was used to identify proteins whose intensity well correlates (positively and negatively) with viscosity, β-glucan, and D-glucose abundance. Fisher’s exact test was used in the enrichment analysis. PANNZER2 [105] and InterProScan [106,107] were used to predict the functions associated with proteins. All the statistical analyses were performed in an R statistical environment (version 3.6.3) [108,109].

The proteome database of *L. brevis* TMW 1.2112 was additionally analyzed by the RAST (rapid annotations using subsystems technology, version 2.0) software [61], eggNOG-Mapper (version 2), a functional annotation tool using the default settings for clusters of orthologous groups (COG), pairwise orthology predictions, and functional annotation [62,63]. Putative signal peptides, which provide an indication of secretory proteins, were identified using SignalP(version 5.0) [66].

### 4.4. Viscosity Analysis

Changes in the viscosity were analyzed daily over the 10-day fermentation period using the rotational viscometer ‘Super 4 Rapid-Visco-Analyser’ (RVA, Perten Instruments, PerkinElmer Company, Waltham, MA, USA) [110,111]. For each measurement, 40 ± 0.01 g of the fermentation broth were weighed in a RVA sample can and analyzed at 20 °C. The RVA configuration was set to 160 rpm with an initial 2 min equilibration phase for a temperature adjustment of the fermentation broth and a subsequent 5 min analysis period under stable conditions. The recorded viscometric data were exported and further evaluated with V. 6.01 GraphPad Prism (GraphPad Software Inc., San Diego, CA, USA). All experiments were carried out using four biological replicates.

### 4.5. Quantification of the β-Glucan and D-Glucose Concentrations

The β-glucan concentration of the supernatant was analyzed by a competitive enzyme-linked immuno-sorbent assay (ELISA) based on *Streptococcus* (*S*.) *pneumoniae* serotype 37 antibodies for the quantification of the bacterial β-glucan [86]. The assay was performed as previously described [36]. The D-glucose concentrations of the supernatant samples were determined using a glucose oxidase/peroxidase assay (GOPOD, Megazyme Ltd., Bray, Ireland) according to the manufacturer’s protocol. The assay was adapted to a microtiter plate volume with a 50 μL sample volume and 150 μL of the GOPOD reagent. A standard curve was used for D-glucose (Megazyme Ltd., Bray, Ireland) calculations. All experiments were carried out using four biological replicates.

## Figures and Tables

**Table 1:** GTs (EC 2.4.-) and GHs (EC 3.2.1.-) enzymes within the genome sequence of *L. brevis* TMW 1.2112 (CP016797).

#	Description	CAZy	EC No.	Gene Locus	Protein Accession Number
1	polysaccharide biosynthesis protein	GT	2.4.1.-.	AZI09_03705	A0A1W6N8G4
2	glycosyltransferase family 2	GT2	2.4.1.-.	AZI09_03685	A0A0C1PWD9
3	glycosyltransferase family 2	GT2	2.4.1.-.	AZI09_12985 ^a^	-
4	glycosyltransferase family 2	GT2	2.4.1.-.	AZI09_07565	A0A1W6NA30
5	glycosyltransferase family 2	GT2	2.4.1.-.	AZI09_06585	A0A1W6N9Q8
6	glycosyltransferase family 2	GT2	2.4.1.-.	AZI09_04045	A0A1W6N8N8
7	glycosyltransferase family 2	GT2	2.4.1.-.	AZI09_12770	A0A1W6NCZ8
8	glycosyltransferase family 2	GT2	2.4.1.-.	AZI09_12875	Q6I7K0
9	glycosyltransferase family 2 (*ykoT*)	GT2	2.4.1.-.	AZI09_10605	A0A1W6NC01
10	exosortase G system-associated	GT2	2.4.1.-.	AZI09_06670	A0A1W6N9Q3
11	glycosyltransferase family 8	GT8	2.4.1.-.	AZI09_12575	A0A1W6NCV8
12	nucleotide-diphospho-sugar transferases superfamily	GT8	2.4.1.-.	AZI09_12410	A0A1W6NCU7
13	glycosyltransferase family 1	GT1	2.4.1.52	AZI09_12995	A0A1W6NDG0
14	poly(glycerol-phosphate) α-glucosyltransferase	GT1	2.4.1.52	AZI09_04905	A0A1W6N8Q2
15	poly(glycerol-phosphate) α-glucosyltransferase	GT1	2.4.1.52	AZI09_04910	A0A1W6N980
16	poly(glycerol-phosphate) α-glucosyltransferase	GT1	2.4.1.52	AZI09_04920	A0A1W6NCH6
17	poly(glycerol-phosphate) α-glucosyltransferase	GT1	2.4.1.52	AZI09_04940	A0A1W6N8Q6
18	poly(glycerol-phosphate) α-glucosyltransferase	GT1	2.4.1.52	AZI09_04945	A0A1W6N8W8
19	UDP glucose-poly(glycerol-phosphate) α-glucosyltransferase (*tagE_6*)	GT1	2.4.1.52	AZI09_04950	A0A1W6N952
20	maltose phosphorylase	GH65	2.4.1.8	AZI09_04670	A0A1W6N8P0
21	maltose phosphorylase	GH65	2.4.1.8	AZI09_01010	A0A1W6N6Y3
22	maltose phosphorylase	GH65	2.4.1.8	AZI09_10320	-
23	endo-β-1,3-glucanase	GH8	3.2.1.-	AZI09_02135 ^a^	-
24	glycosyl hydrolase family 18	GH18	3.2.1.14	AZI09_03025 ^a^	A0A1W6NI26
25	glycosyl hydrolase family 88	GH88	3.2.1.172	AZI09_11545	A0A1W6NC28
26	glycosyl hydrolase family 31	GH31	3.2.1.177	AZI09_02865	A0A1W6N888
27	α-xylosidase	GH31	3.2.1.177	AZI09_09820	A0A1W6NBL2
28	glucohydrolase	GH13	3.2.1.10	AZI09_00950	A0A1W6N750
29	glucohydrolase (*malL_2*)	GH13	3.2.1.10	AZI09_10575	A0A1W6NBX6
30	α-glucosidase	GH31	3.2.1.20	AZI09_08630	A0A1W6NAK3
31	α-glucosidase	GH31	3.2.1.20	AZI09_11465	A0A1W6NBZ8
32	α-glucosidase	GH31	3.2.1.20	AZI09_12510	A0A1W6NCT8
33	β-1,3-glucosidase (*bglB*)	GH3	3.2.1.21	AZI09_02170	A0A1W6N7S3
34	xylan 1,4-β-xylosidase	GH39	3.2.1.37	AZI09_11985	A0A1W6NC86
35	β-xylosidase (*xynB*)	GH43	3.2.1.37	AZI09_11935	A0A1W6NCN2
36	glucosylceramidase	GH30	3.2.1.45	AZI09_00755	A0A1W6N733
37	α-L-arabinofuranosidase 1	GH51	3.2.1.55	AZI09_03165	A0A1W6N7 × 2
38	α-N-arabinofuranosidase (*abfA_1*)	GH51	3.2.1.55	AZI09_00785	A0A1W6N6T5
39	6-phospho-β-glucosidase	GH1	3.2.1.86	AZI09_09805	A0A1W6NB21
**GHs and GTs Associated with Cell Wall Biosynthesis**					
40	DD-transpeptidase	GT51	3.4.16.4	AZI09_04800	A0A1W6N956
41	penicillin-binding protein	GT51	2.4.1.129	AZI09_06425	A0A1W6N9H6
42	N-acetylglucosaminyldiphosphoundecaprenol N-acetyl-beta-D-mannosaminyltransferase	GT26	2.4.1.187	AZI09_09370	A0A1W6NAY4
43	UDP-N-acetyl-D-mannosamine transferase	GT26	2.4.1.187	AZI09_03665	A0A1X0XQ74
44	poly(glycerol-phosphate) α-glucosyltransferase	GT1	2.4.1.208	AZI09_04170	A0A1W6N8F4
45	1,2-diacylglycerol 3-glycosyltransferase (*pimA*)	GT1	2.4.1.337	AZI09_04165	A0A1W6N8G5
46	N-acetylglucosaminyltransferase (*murG*)	GT28	2.4.1.227	AZI09_05030	A0A1W6N9A0
47	N-acetylmuramide glycanhydrolase	GH25	3.2.1.-	AZI09_10600 ^a^	A0A1W6NBH8
48	N-acetylmuramoyl-L-alanine amidase	GH73	3.2.1.96	AZI09_04775 ^a^	A0A1W6N923
49	N-acetylmuramoyl-L-alanine amidase (*atl_1*)	GH73	3.2.1.96	AZI09_02505 ^a^	A0A1W6N829

^a^ Putative signal peptide proteins.

## Data Availability

The mass spectrometry proteomics data have been deposited to the ProteomeXchange Consortium (http://proteomecentral.proteomexchange.org) via the PRIDE partner repository with the dataset identifier PXD031809.

## Supplementary Materials

The following supporting information can be downloaded at: https://www.mdpi.com/article/10.3390/ijms23063393/s1.
