# Supplementary figures and images for: Proteomic Analysis Reveals Enzymes for β-D-Glucan Formation and Degradation in Levilactobacillus brevis TMW 1.2112
Source: Int J Mol Sci. 2022 Mar 21;23(6):3393. doi: 10.3390/ijms23063393 (PMC8951740; doi:10.3390/ijms23063393)

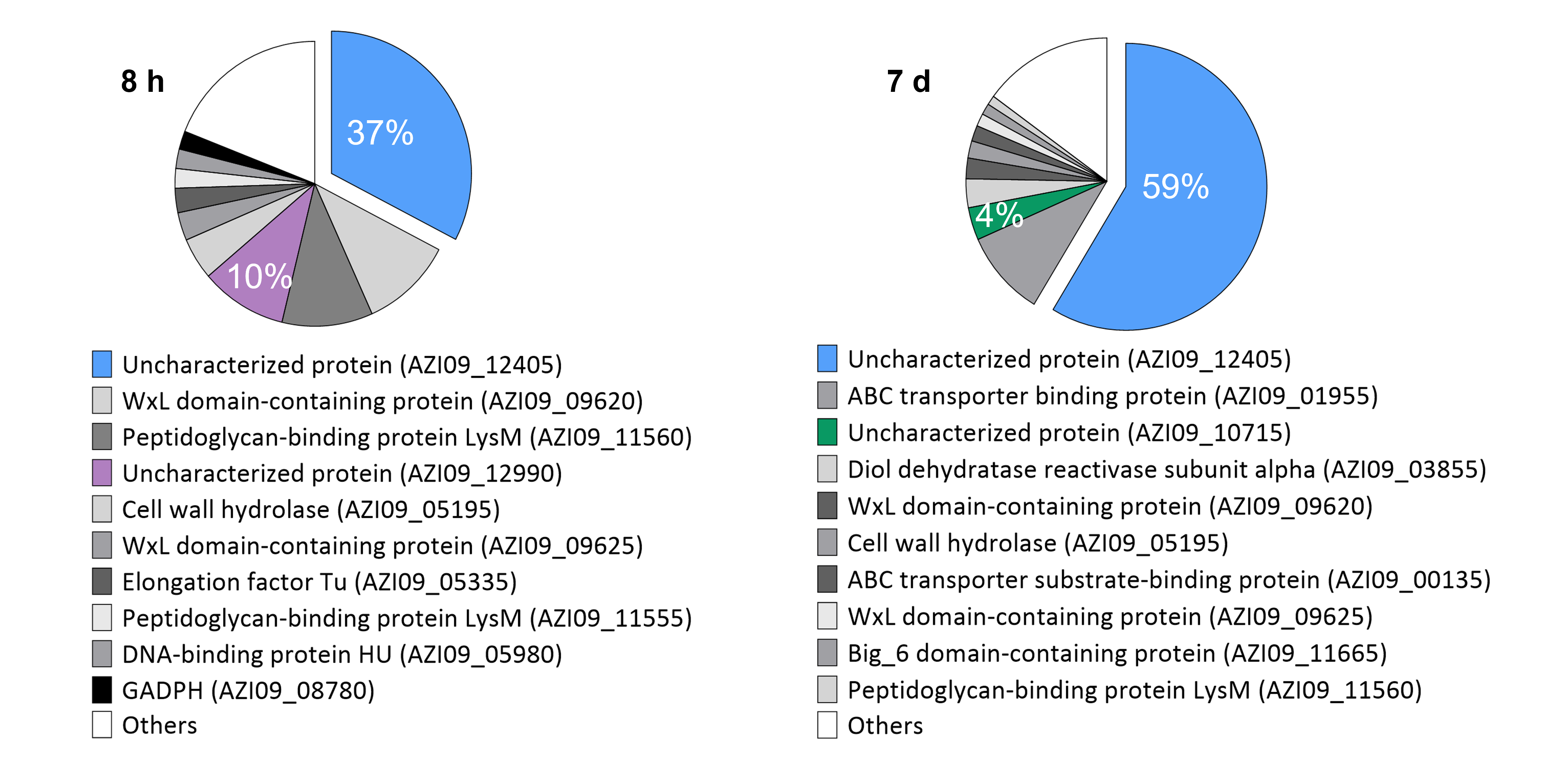

Supplement: Supplementary file 1 [file ijms-23-03393-s001.zip › Figure S1.tif]
